# Biotransformation and Oxidative Stress Responses in Captive Nile Crocodile (*Crocodylus niloticus*) Exposed to Organic Contaminants from the Natural Environment in South Africa

**DOI:** 10.1371/journal.pone.0130002

**Published:** 2015-06-18

**Authors:** Augustine Arukwe, Randi Røsbak, Aina O. Adeogun, Håkon A. Langberg, Annette Venter, Jan Myburgh, Christo Botha, Maura Benedetti, Francesco Regoli

**Affiliations:** 1 Department of Biology, Norwegian University of Science and Technology (NTNU), 7491 Trondheim, Norway; 2 Department of Zoology, University of Ibadan, Ibadan, Nigeria; 3 Department of Paraclinical Sciences, Faculty of Veterinary Science, University of Pretoria, Private Bag X04, Onderstepoort, 0110, South Africa; 4 Dipartimento di Scienze della Vita e dell’Ambiente, Università Politecnica delle Marche, Ancona, Italy; The University of Iowa, UNITED STATES

## Abstract

In the present study, the biotransformation and oxidative stress responses in relation to chemical burden in the liver of male and female Nile crocodiles—*Crocodylus niloticus*—from a commercial crocodile farm passively exposed to various anthropogenic aquatic pollutants was investigated. In general, the data showed that male crocodiles consistently produced higher biotransformation and oxidative stress responses compared to females. Relationships between these responses and concentrations of aliphatic hydrocarbons and polycyclic aromatic hydrocarbons (PAHs) were also observed. Specifically, the catalytic assays for EROD and BROD (not PROD and MROD) showed sex-differences between male and female crocodiles and paralleled immunochemically determined CYP1A and CYP3A protein levels; the relatively similar levels of PAHs in both sexes suggest an estrogen-mediated reduction of this pathway in females. The antioxidant system exhibited higher levels in male crocodiles with slight or significant higher values for catalase (CAT), glutathione reductase (GR), glutathione peroxidases-H_2_O_2_ (GPx-H_2_O_2_), glutathione peroxidases-Cu (GPx-Cu), total antioxidant capacity towards peroxyl radicals (TOSC-ROO) and hydroxyl radicals (TOSC-HO), total glutathione (GSH) and malondialdehyde (MDA). On the other hand, the activities of acyl-CoA oxidase (AOX) and glutathione S-transferases (GST) were significantly higher in females. Principal component analysis (PCA) produced significant groupings that revealed correlative relationships (both positive and negative) between biotransformation/oxidative stress variables and liver PAHs and aliphatic hydrocarbon burden. The overall results suggest that these captive pre-slaughter crocodiles exhibited adverse exposure responses to anthropogenic aquatic contaminants with potentially relevant effects on key cellular pathways, and these responses may be established as relevant species biomarkers of exposure and effects in this endangered species.

## Introduction

The release of environmental contaminants, including polycyclic aromatic hydrocarbons (PAHs), pharmaceuticals and other persistent organic pollutants (POPs) to the terrestrial and aquatic environments has been of concern for several decades [1–3]. Environmental contaminants, including PAHs and pharmaceuticals may be excreted both unmetabolized or as metabolites [4,5]. Exposure of an organism to xenobiotics activates biological processes in order to metabolize and eliminate these foreign chemicals through a process generally referred to as biotransformation [6]. Biotransformation activities are divided into phase 1 and 2 reactions [7]. Phase 1 reactions result in loss of pharmacological activity of the xenobiotics through the introduction or exposure of a functional group on the parent compound [8], while phase 2 reactions then introduce a covalent linkage between the functional group and an endogenous water soluble conjugate such as glucuronic acid to facilitate excretion [9]. Once conjugated, the xenobiotics become more soluble and are easily excreted. The cytochrome P450 (or CYP) enzyme systems play an integral role in the biotransformation process [10].

In biomonitoring studies, the activity of CYP enzymes can provide an indication of their presence in an environmental matrice or biota [11]. In the aquatic ecosystem, the induction of CYP1A measured as 7-ethoxyresorufin-O-deethylase (EROD activity or CYP1A transcripts) has been widely used as a sensitive and convenient early warning system of biological exposure to organic pollutants [11]. Several classes of PAHs and POPs also act as prooxidant stressors increasing the intracellular generation of reactive oxygen species (ROS) causing oxidative conditions which in turn modulate levels and functions of redox-sensitive signaling proteins and antioxidants [12]. A group of cytoprotective enzymes such as the glutathione transfersase (GST), epoxide hydrolase (EH), NAD(P)H:quinone oxidoreductase 1 (NQO1), uridine diphospho-glucuronosyltransferase (UDPGT), aldehyde dehydrogenase 1A1, aldo-keto reductase, glutathione reductase, catalase, Cu/Zn-superoxide dismutase (SOD) and glutathione peroxidases (GPx) that are coded through the antioxidant responsive element (ARE) form a network of protective machinery against oxidative stress [12–15]. Unlike the CYP systems, changes in the antioxidant responses are generally difficult to predict. Induction, inhibition, biphasic or temporary changes can be observed depending on experimental conditions, intensity and duration of exposure, species or tissues used, metabolic status or presence of other confounding factors have been reported [13–15]. To our knowledge, these biochemical responses to environmental stressors have not been studied in the Nile crocodile.

The Nile crocodile is highly exposed to anthropogenic contaminants in its natural habitats. Uncontrolled discharges of domestic and industrial wastes pose a serious threat to this endangered species. Indeed, past studies have reported high concentrations of metals and other environmental organic pollutants in selected tissues of *C*. *niloticus* [16]. However, little is known about sublethal effects from exposure of *C*. *niloticus* to environmental contaminants through the analysis of biomarkers directly related to detoxification or adaptation processes. The distribution of the Nile crocodile covers about 75% of the African continent [17]. This species can be found in a variety of habitats that include lakes, rivers, freshwater swamps and brackish water. Juvenile Nile crocodiles change from feeding on small aquatic invertebrates to larger vertebrates that include fish, amphibians and reptiles. While the adult crocodiles are capable of feeding on larger vertebrates like buffalo and antelope, fish and smaller vertebrates comprise a large part of their diet [17]. The Nile crocodile is the largest of the 3 crocodile species found in Africa and is the best known of all of the crocodile species occurring throughout most of Africa and Madagascar. They are mostly aquatic, but travel easily on land. Treated sewage water or other wastewater from cities, towns or villages are linked to the environmental release of chemicals such as PAHs, pharmaceutical agents, POPs and industrial by-products [18,19]. Therefore, the aims of the present study were to (1) evaluate the expression of molecular and biochemical responses in the liver of farmed Nile crocodile exposed to potentially polluted water in the breeding dams, (2) determine potential sex-differences in these molecular and biochemical responses, (3) evaluate these responses in relation to level of contamination in liver, and (4) establish the usefulness of these responses as biomarkers of exposure and effect in this endangered species.

## Materials and Methods

### Chemicals and reagents

Bovine serum albumin (BSA), NADPH, 1-chloro-2,4-dinitrobenzene (CNDB), glutathione, o-phenylenediamine dihydrochloride (OPD), ethoxyresorufin, methoxyresorufin, benzyloxyresorufin and pentoxyresorufin, were purchased from Sigma-Aldrich Chemical Co. (St. Louis, MO). Microtiter plates (MaxiSorp) were purchased from Nunc (Roskilde, Denmark). Polyclonal CYP1A1 antibody was purchased from Biosense Laboratories, (Bergen, Norway). CYP2B6, CYP2E1 and CYP2B1/2 antibodies were purchased from Santa Cruz Biotechnology (Dallas TX, USA). The CYP3A antiserum was a kind gift from Dr. Malin Celander. All other chemicals were of the highest commercially available grade.

### Crocodile tissue sampling

**Fig 1:**
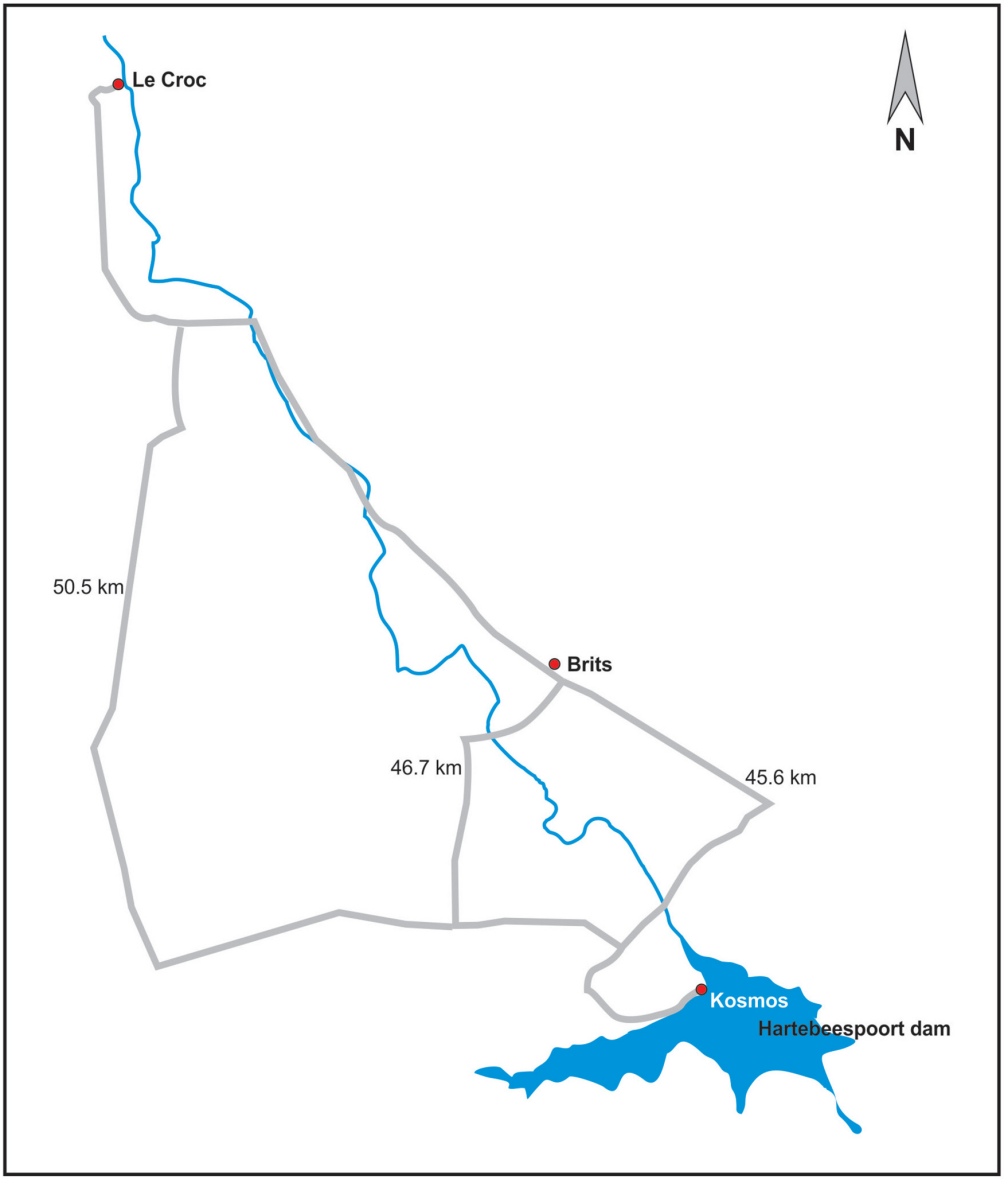
Map of the outflow water from the Hartbeespoort Dam through the Crocodile River-West in a northern direction, passing the sampling site (Le Croc farm) and on its eastern border, and then to the Limpopo River that is the border between South Africa and Botswana as well as Zimbabwe.

The study animals were sampled at Le Croc farm, which is located downstream of the Hartbeespoort dam and the sewage treatment plant (STP) in the town of Brits (about 40 km West of Pretoria) South Africa. The Hartbeespoort dam is the primary recipient of all sewage and wastewater outflow from Johannesburg and surrounding towns. The outflow water of the Hartbeespoort Dam flows in the Crocodile River-West in a northern direction, passing the crocodile farm on its eastern border, and then to the Limpopo River (border between South Africa and Botswana as well as Zimbabwe: Fig 1). Thus, Le Croc farm is next to the Crocodile river in the Northwest Province of South Africa; 20 km north of Brits town, which is situated in the Northwest Province of South Africa. GPS location is: S 25 29.548, E 027 40.827. Water for the Le Croc farm is obtained directly from the Crocodile-West River. Tissues (gonad, liver, plasma, adipose tissue and kidney) from 14 male and 15 female crocodiles were sampled during periodic slaughtering at the farm abattoir. The liver samples were only used in this report, because this organ is the central for metabolic and synthetic processes, including phase I and II biotransformation reactions. The crocodiles used in this study were approximately 2 years old, and total weight and length did not differ significantly between males and females. It should be noted that this study was initiated because of the persistent general health and reproductive problems that have been report at Le Croc farm in recent years. The crocodiles were given electro shock, thereafter pithing was applied prior to decapitation. The tissues were collected during a routine slaughtering at Le Croc farms with permission from the management, labelled and snap-frozen in liquid nitrogen and transported to the laboratory at the Department of Paraclinical Sciences, Faculty of Veterinary Science, University of Pretoria at Onderstepoort, and was shipped to Trondheim thereafter.

The experiments/procedures described in our paper are conducted in accordance with the laws and regulations controlling experiments with life animals in South Africa (where the experiment was conducted). Given that the crocodiles were not sacrificed for the sole purpose of the present study, and the tissue samples that otherwise would have gone to waste, were collected during routine slaughtering at Le Croc farms, no other approved by an Institutional Animal Care and Use Committee (IACUC) or equivalent animal ethics committee was needed. However, Le Croc farms operate under strict South African laws with a committee that oversees the ethical care and sacrifice of the animals. In addition, all sampling procedures were reviewed or specifically approved as part of obtaining the field study permit.

### Chemical analyses

Aliphatic hydrocarbons (C10-C40), polycyclic aromatic hydrocarbons (PAHs), polychlorinated biphenyls (PCBs), organo-halogenated pesticides (OCPs), chlorophenols, monoaromatic compounds (BTEX: benzene, toluene, ethylbenzene and xylene congeners), brominated flame retardants and trace metal were analyzed in crocodile liver by conventional procedures based on gas-chromatography with flame ionization detector, electron capture detector and mass detector, high performance liquid chromatography (HPLC) with diode array and fluorimetric detection, atomic absorption spectrophotometry. Details on analytical methods and procedures for quality assurance/quality control are given in Supplementary Material (S1 File).

### Biomarkers analyses

Biomarkers were measured in the liver through standardized protocols which included: spectrofluorimetric analysis of ethoxyresorufin *O*-deethylase (EROD), pentoxyresorufin *O*-deethylase (PROD), buthoxyresorufin *O*-deethylase (BROD) and methoxyresorufin *O*-deethylase (MROD) activities, spectrophotometric determination of acyl-CoA oxidase (AOX), antioxidants defences (catalase, GST, Se-dependent and sum of Se-dependent and Se-independent glutathione peroxidases, GPx-H_2_O_2_ and GPx-CHP, GR, glutathione: GSH), malondialdehyde (MDA); gas-chromatographic assay of total antioxidant capacity (TOSC) towards peroxyl radicals (TOSC-ROO·) and hydroxyl radicals (TOSC-HO·). Detailed protocols are given in S1 File.

### Immunochemical analysis

Total liver microsomal protein concentrations in samples were determined with the Bradford method [20] using bovine serum albumin (BSA) as standard. Total cellular proteins (50 μg) were separated by 12% precast sodium dodecyl sulfate polyacrylamide gel electrophoresis (SDS-PAGE: Bio-Rad). The gel was blotted onto polyvinylidene difluoride (PVDF) membranes (Bio-Rad) and incubated with the primary polyclonal antibodies: CYP1A (Biosense Laboratories 1:500), CYP3A (1:2000), and CYP2E1, CYP2B1/2B2, CYP26 (Santa Cruz Biotechnologies, 1:500) antisera. After washing, membranes were incubated with peroxidase conjugated goat anti-rabbit/mouse antibodies (GAR/GAM-HRP; Bio-Rad) diluted 1:3000, developed using an Immun-Star Western Chemiluminescent Kit (Bio-Rad) and visualized with Eastman Kodak Company’s Molecular Imaging Systems (Rochester, NY, USA).

### Statistical analyses

All data are presented as mean ± standard error of the mean (SEM). Statistical analyses of enzyme activities were performed using SPSS statistical software, version 20. Normality within each group was tested using the Shapiro-Wilk test. Possible single outliers were detected and assessed using box and whiskers plot provided by SPSS combined with Grubbs’ test for outliers. By using power transformations, non-normal datasets was attempted to approach normality. As normal distribution within groups was achieved, datasets were tested for homoscedasticity using Levenes test. Given homoscedasticity, group means for males and females was tested using one-way ANOVA followed by student t-test to detect significant differences between males and females. Chemical data together with biochemical and oxidative stress parameters results were analyzed using Principal component analysis (PCA). Possibly correlated variables were evaluated using Spearman's Rank-Order Correlation in SPSS. PCA analyses were performed using Umetrics SIMCA-P^+^ software version 12.01. Significance level was set to α = 0.05. Graphs were made using Systat Software SigmaPlot, version 12.5.

## Results

**Fig 2:**
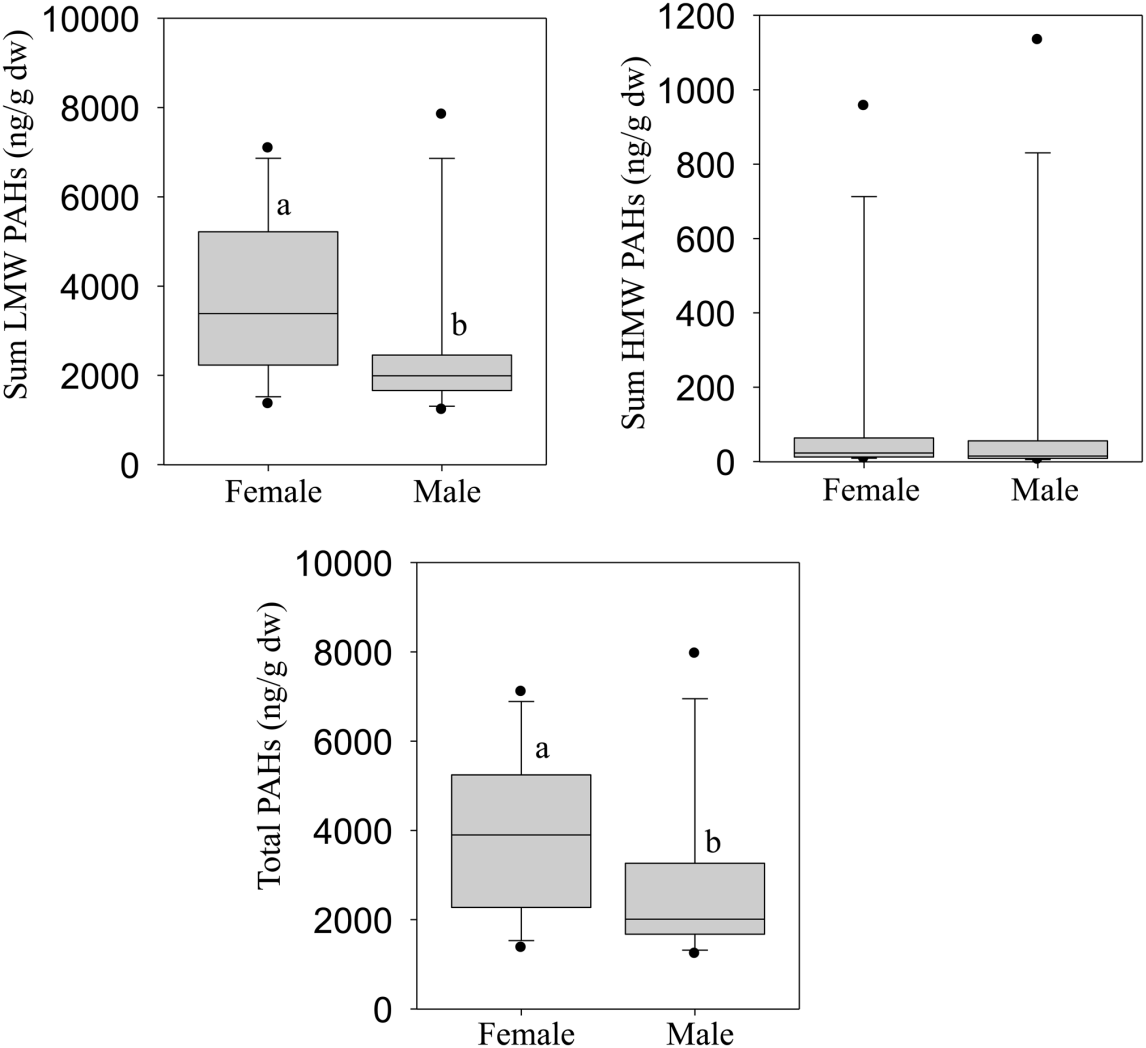
Liver concentrations of sum low molecular weight (LMW: A), sum high molecular weight (HMW: B) and total (C) polyaromatic hydrocarbons (PAHs) in female and male Nile crocodiles (*Crocodylus niloticus)* from a commercial crocodile farm (Le Croc) in Pretoria South Africa. All values represent the mean (n = 14 females or males) ± standard error of the mean (SEM). Different letters denote significant differences (p<0.05) between females and males, analyzed using student t-test.

All chemical classes were analyzed in both male and female crocodiles and the entire set of analytical results are provided in Supplementary Material (Table A in S1 Dataset and Fig. A in S1 Dataset). Total aliphatic hydrocarbons did not exhibit significant differences between males and females with concentrations of 5900 ± 4500 and 4200 ± 2900 μg/g dry weight (dw: mean ± standard deviation) respectively (Table A in S1 Dataset and Fig. A in S1 Dataset). The dominant groups of these hydrocarbons were C30-C32 > C20-C22 > C18-C20 > C32-C34 with a similar distribution in both sexes; the C30-C32 compounds were measured at 2604 ± 752 μg/g dw in males, and 2900 ± 840 μg/g dw in female crocodiles (Table A in S1 Dataset and Fig. A in S1 Dataset). Among PAHs, the low-molecular weight (LMW; Fig 2A) hydrocarbons were significantly higher in female crocodiles, while no sex-related differences were observed for the sum of high molecular weight (HMW; Fig 2B) PAHs despite some variations in individual compounds (Table A in S1 Dataset and Fig. A in S1 Dataset). Total PAHs ranged from 3900 ± 1820 μg/g dw in female to 2900 ± 1940 μg/g dw in male crocodiles (Fig 2C). Concentrations of monoaromatic hydrocarbons (BTEX) were not significantly different between male and female crocodile livers with respective 3690 ± 1685 and 3233 ± 940 μg/g dw. However, these values were almost exclusively represented by benzene. Polychlorinated biphenyls (PCBs), organo-halogenated pesticides (OCPs) and chlorophenols were generally below the detection limit, except for few individual compounds with quantifiable levels including, 2,4,6-trichlorophenol, 2,4-dichlorophenol, pentachlorophenol, heptachlor, heptachlor epoxide, endosulfan I and endosulfan II (Table B in S1 Dataset). Variable results were obtained for brominated flame retardants with values ranging from less than 5 ng/g dw to more than 640 ng/g dw; PBDE 153, 154, 183 were the most represented compounds (Table B in S1 Dataset).

Trace metals did not differ between male and female crocodiles with mean average values of 0.2 μg/g dw for As, 0.1 μg/g dw for Ba, 0.1 μg/g dw for Cd, 0.4 μg/g dw for Cr, 22 μg/g dw for Cu, 1900 μg/g dw for Fe, 0.1 μg/g dw for Hg, 2 μg/g dw for Mn, 0.2 μg/g dw for Ni, 0.3 μg/g dw for Pb, 1.3 μg/g dw for V, 41 μg/g dw for Zn (Table B in S1 Dataset).

### Biotransformation biomarker responses

**Fig 3:**
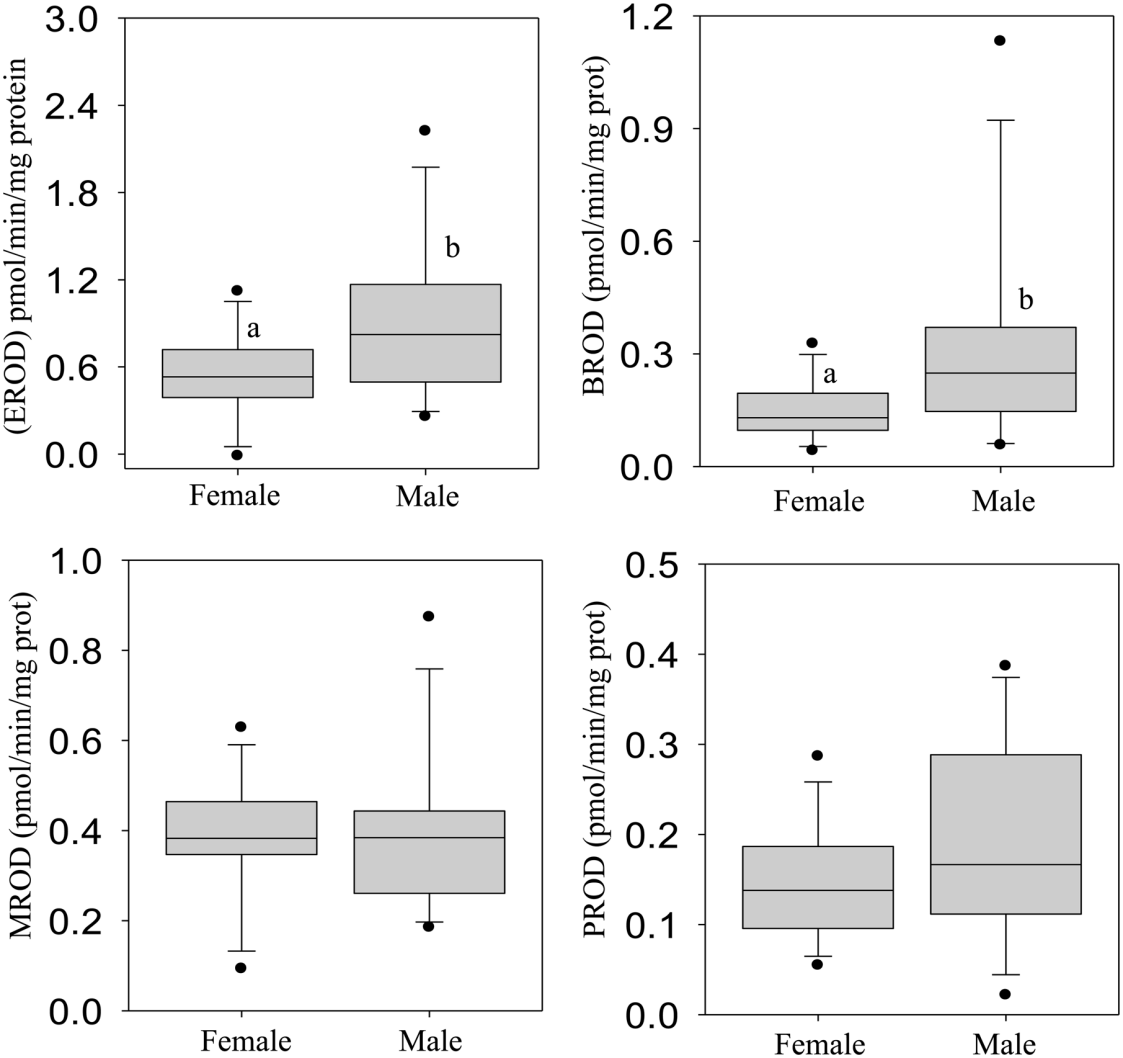
Cytochrome P450-mediated catalytic ethoxyresorufin (EROD: A), benzyloxyresorufin (BROD: B), methoxyresorufin (MROD: C) and pentoxyresorufin (PROD: D) activities in female and male Nile crocodile (*Crocodylus niloticus)* from a commercial crocodile farm (Le Croc) in Pretoria, South Africa exposed to various anthropogenic aquatic pollutants. All values represent the mean (n = 14 females or males) ± standard error of the mean (SEM). Different letters denote significant differences (p<0.05) between females and males, analyzed using student t-test.

**Fig 4:**
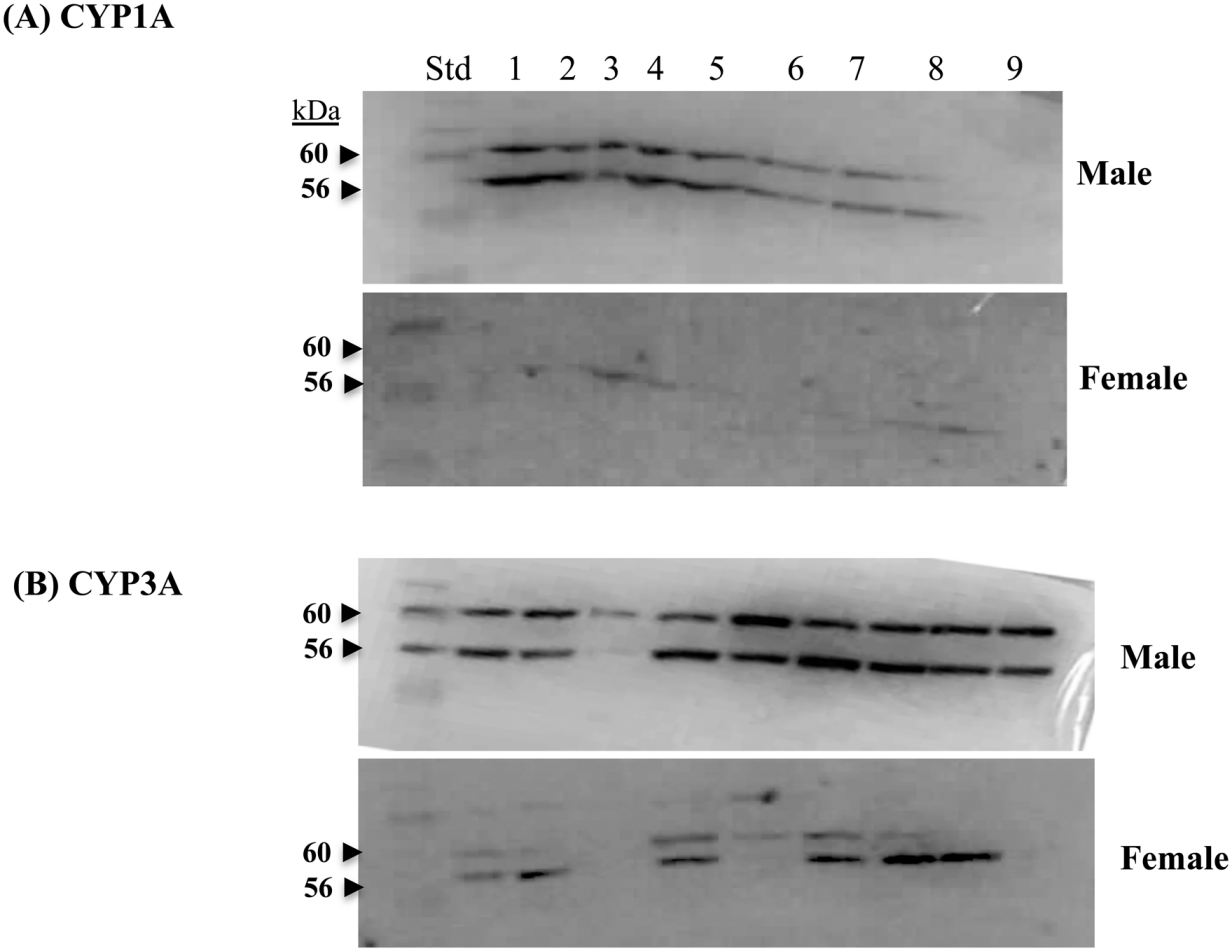
Immunochemical detection using Western blotting of CYP1A (A) and CYP3A (B) proteins in female and male Nile crocodile (*Crocodylus niloticus)* from a commercial crocodile farm (Le Croc) in Pretoria, South Africa exposed to various anthropogenic aquatic pollutants. 20 μg of total hepatic protein was loaded per well.

The activities of xenobiotic biotransformation enzyme systems showed that hepatic EROD (CYP1A1) and BROD (CYP1A, 2B, 3A) activities were significantly higher in male crocodiles, compared to females (Fig 3A and 3B, respectively). The MROD (CYP1A2) and PROD (CYP2B) activities did not show any significant sex-related differences (Fig 3C and 3D, respectively). Similarly, immunochemical analysis of CYP1A and CYP3A proteins indicated cross-reactions, with male crocodiles expressing higher protein band intensities, compared to females (Fig 4A and 4B, respectively). However, immunoblot analysis of CYP2B, CYP2E and CYP26 proteins did not reveal detectable protein cross-reactions in both male and female crocodiles (data not shown).

### Oxidative stress biomarker responses

**Fig 5:**
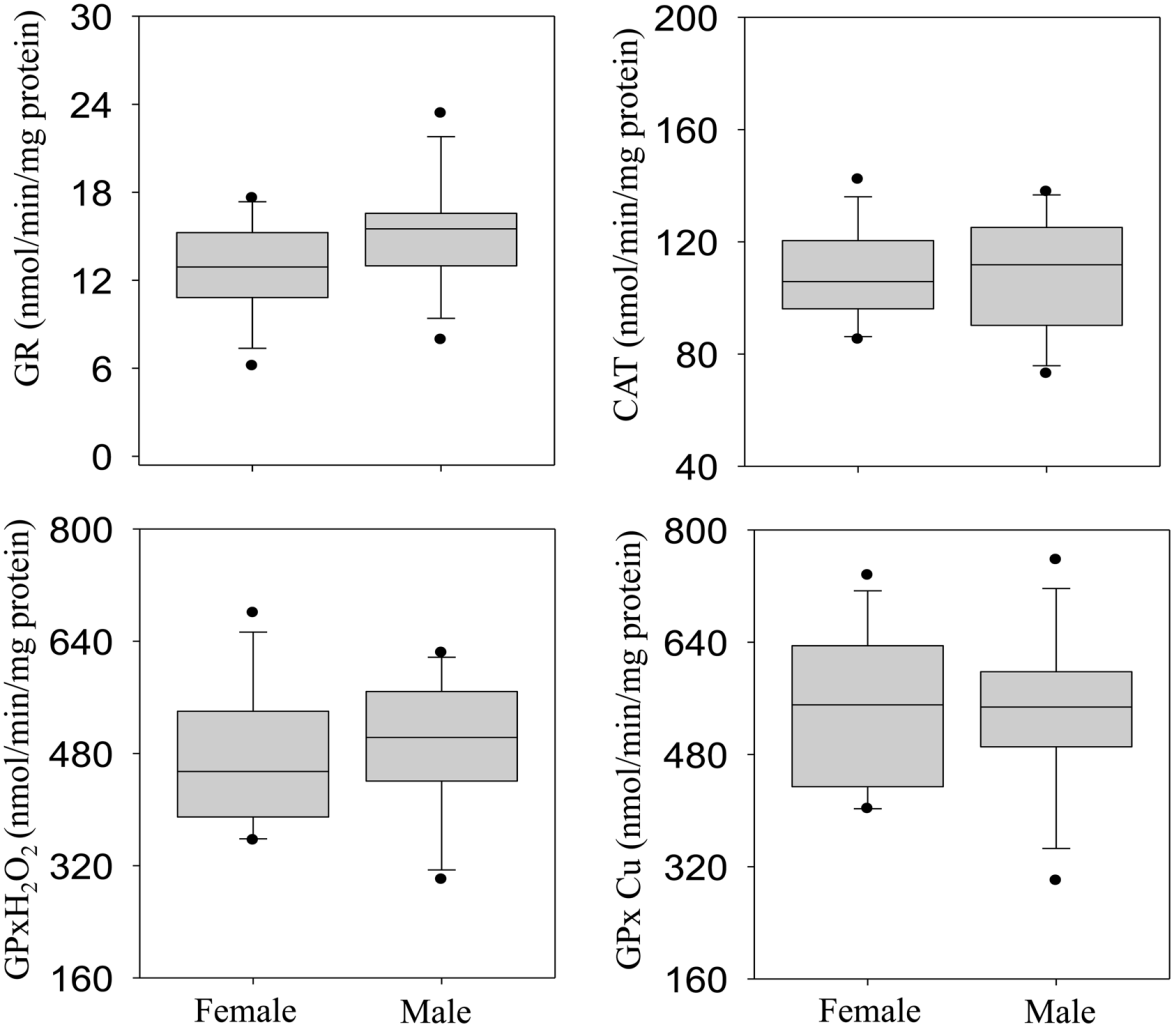
Liver activities of glutathione reductase (GR: A), catalase (CAT: B), glutathione peroxidases-H_2_O_2_ (GPx-H_2_O_2:_ C), glutathione peroxidases-Cu (GPx-Cu; D) in female and male Nile crocodile (*Crocodylus niloticus)* from a commercial crocodile farm (Le Croc) in Pretoria, South Africa exposed to various anthropogenic aquatic pollutants. All values represent the mean (n = 14 females or males) ± standard error of the mean (SEM). Different letters denote significant differences (p<0.05) between females and males, analyzed using student t-test.

**Fig 6:**
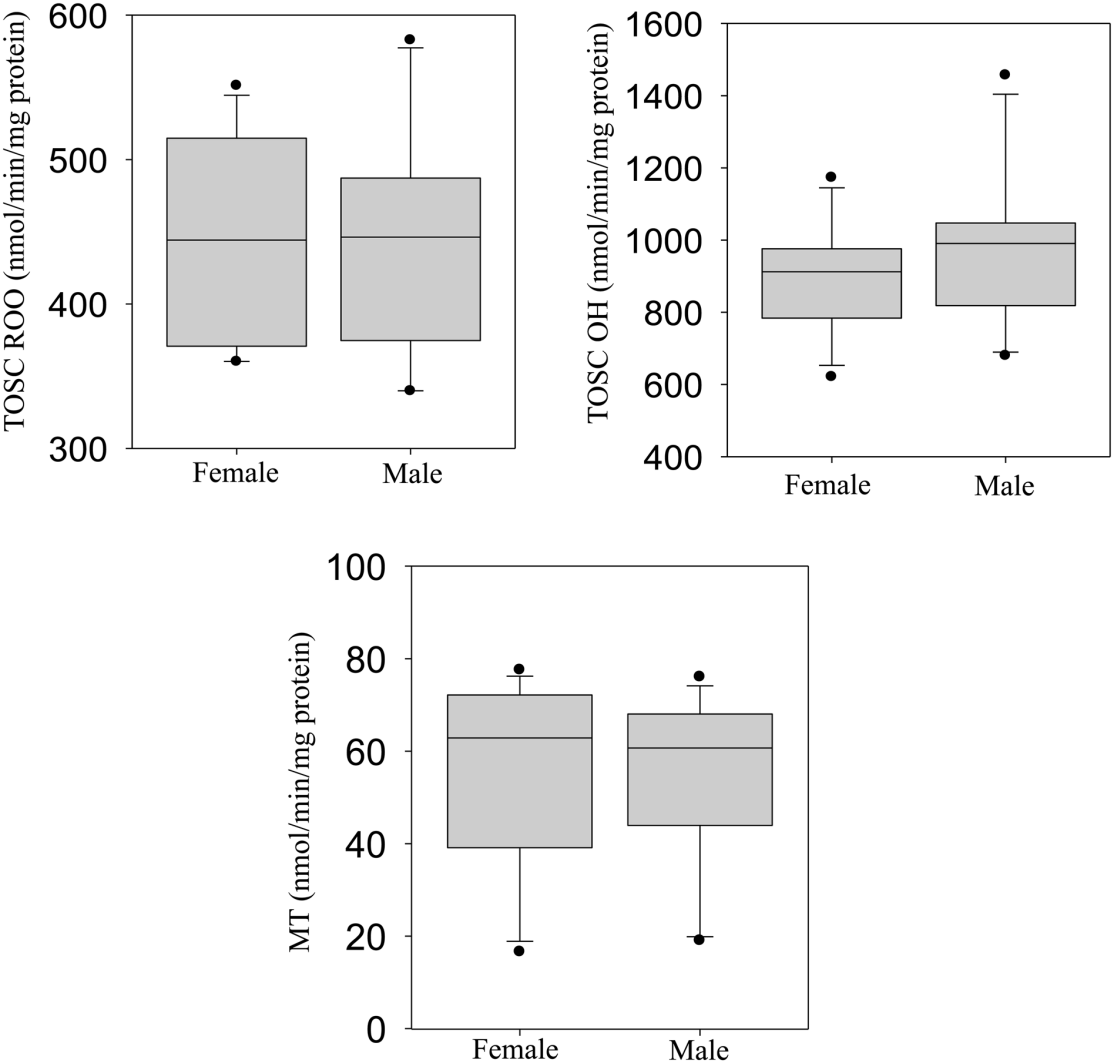
Liver levels of total antioxidant capacity towards peroxyl radicals (TOSC-ROO·: A), hydroxyl radicals (TOSC-HO·: B) and metallothionein (MT) in female and male Nile crocodile (*Crocodylus niloticus)* from a commercial crocodile farm (Le Croc) in Pretoria, South Africa exposed to various anthropogenic aquatic pollutants. All values represent the mean (n = 14 females or males) ± standard error of the mean (SEM). Different letters denote significant differences (p<0.05) between females and males, analyzed using student t-test.

**Fig 7:**
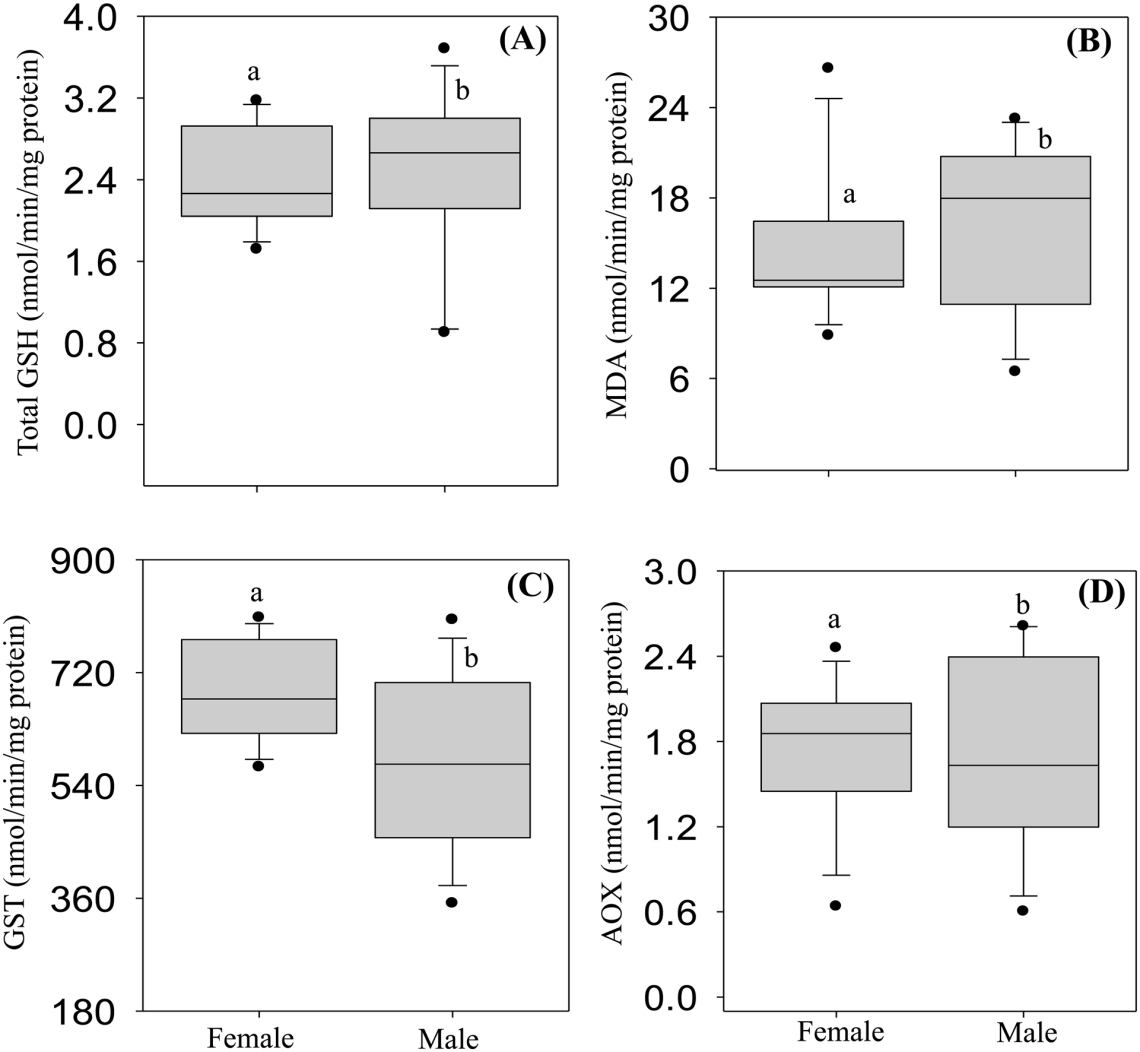
Liver levels of total glutathione (GSH: A), malondialdehyde (MDA: B), glutathione S-transferases (GST: C) and acyl-CoA oxidase (AOX: D) in female and male Nile crocodile (*Crocodylus niloticus)* from a commercial crocodile farm (Le Croc) in Pretoria, South Africa exposed to various anthropogenic aquatic pollutants. All values represent the mean (n = 14 females or males) ± standard error of the mean (SEM). Different letters denote significant differences (p<0.05) between females and males, analyzed using student t-test.

The majority of antioxidant enzymes (GR, GPx-H_2_O_2_, GPx-Cu and CAT) and the total oxyradical scavenging capacity (TOSC-ROO· and TOSC-HO·) were slightly higher in male than female crocodiles, albeit the magnitude of such differences was not statistically significant (Figs 5 and 6). Liver metallothionein (MT) level was not significantly different between male and female crocodiles (Fig 6C). In addition, males exhibited significantly higher activities of GSH and MDA (Fig 7A and 7B, respectively), while GST and AOX activities were significantly higher in females (Fig 7C and 7D, respectively).

### Multivariate data analysis

**Fig 8:**
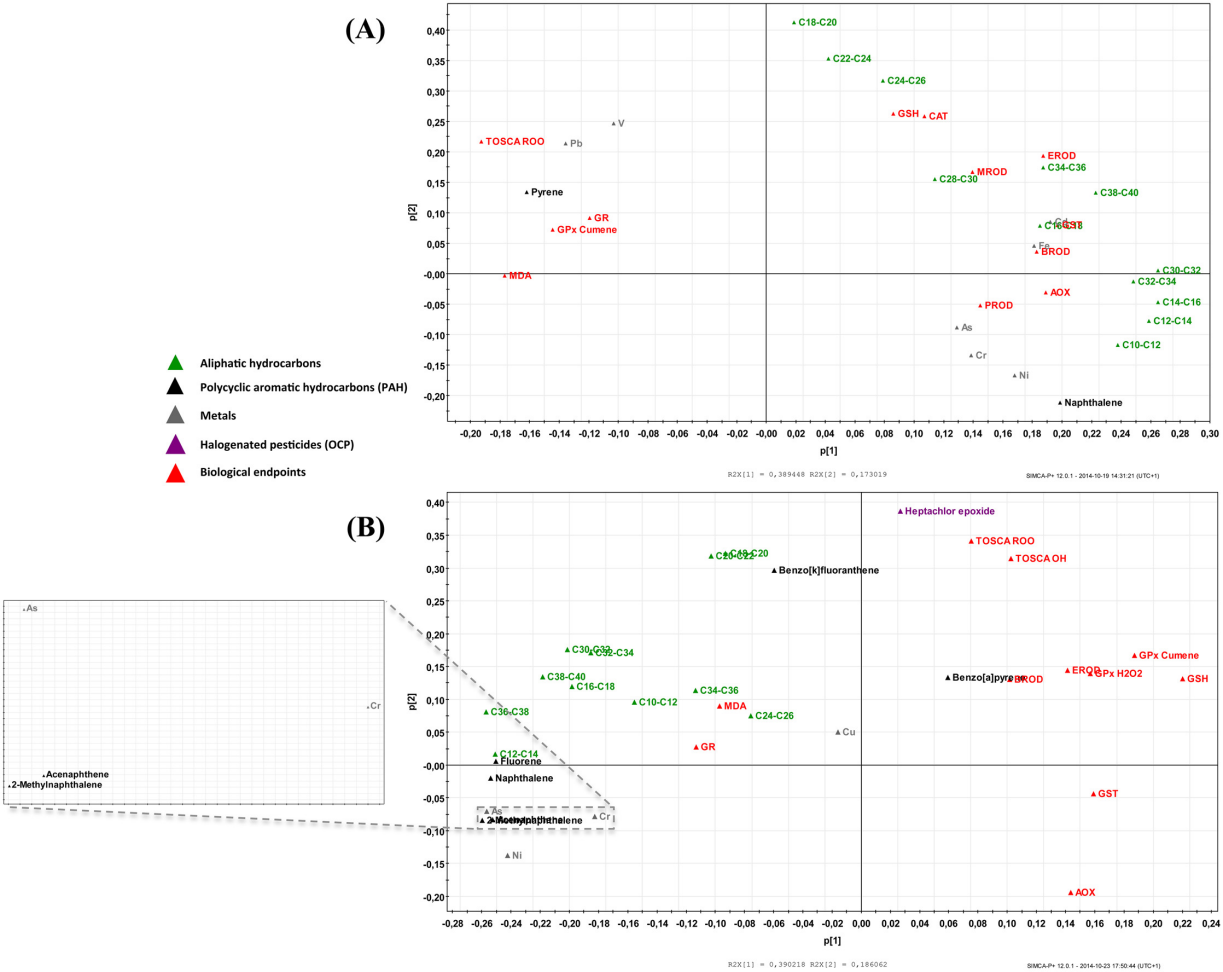
Principal component analysis (PCA) showing individual contaminant and variable loadings according to liver chemical burden and oxidative stress/biotransformation variables in females (A) and male (B) Nile crocodile (*Crocodylus niloticus)* from a commercial crocodile farm (Le Croc) in Pretoria, South Africa exposed to various anthropogenic aquatic pollutants.

Loading plots of principal component analysis (PCA) produced significant grouping of individual contaminant and variable loadings according to liver chemical burden and oxidative stress/biotransformation variables. Overall, GPx-Cu, GR, TOSCA ROO and MDA were situated opposite to the aliphatic hydrocarbons and LMW PAHs in females and showed significant negative relationships (Fig 8A); the same variables were separated from HMW PAHs along PC2 and from AOX along PC1 in female crocodiles (Fig 8A). On the other hand, BROD, CAT, EROD, GSH and PROD showed significant positive relationship with aliphatic hydrocarbons in females (Fig 8A). For the males, GSH, GST and AOX were situated opposite to the aliphatic hydrocarbons and LMW PAHs and showed significant negative relationships (Fig 8B). On the other hand, EROD and GR showed significant positive relationship with BaP and aliphatic hydrocarbons, respectively, in males (Fig 8B).

## Discussion

In most parts of Africa, surface water is often utilized as drinking water and in food preparation, without prior and suitable purification. In addition, aquatic species are often consumed and form a large part of the diet of rural populations in Africa. Thus, tissue residues in the flesh contribute to the daily intake of contaminants by humans [21] and also represent serious health consequences for biota [22]. In the present study, the biotransformation and oxidative stress responses in relation to chemical burden in the liver of male and female Nile crocodiles was investigated, with varying degrees of anthropogenic chemical contamination. Among the analyzed chemicals, polychlorinated biphenyls (PCBs), organo-halogenated pesticides (OCPs) and brominated flame retardants were generally present at lower concentration compared to those previously reported in alligators from China [23] and Florida [24–26]. Trace metals concentrations were comparable to those reported in crocodiles (*C*. *niloticus*) from Zambia and American alligators—*Alligator mississippiensis* [24,27]. On the other hand, concentrations of aliphatic hydrocarbons, PAHs and benzene (among the monoaromatic hydrocarbons) exhibited relatively high values compared to those normally present in aquatic vertebrates [28]. Overall, the reported data are real, with high error values that are typical for field samples. However, in the absence of Nile crocodile historical biotransformation or oxidative stress data, our data does not directly show cause and effect, but show strong relationship between tissue contaminants burden and biological responses, and directly correlates with the current reproductive and general health problems that is being observed at Le Croc farm. Therefore, the present study represents a solid basis for understanding the potential physiological, reproductive and general health consequences of exposure of this threatened species to contaminants in their natural environment.

### Phase I and II biotransformation responses

Hepatic and extra-hepatic CYPs in several species, including mice and rats, exhibit sex-specific expression patterns [29–31], suggesting a possible regulatory role by sex steroids. For example, the post-pubertal expression of several sex-specific CYP forms are known to be regulated by gonadal steroids through neonatal programming [31]. To our knowledge, there are no studies or historical report that have investigated the activities and protein levels of CYP isoforms in the Nile crocodile, either during a reproductive cycle or in relation to the potential influence of environmental contaminants. Captive Nile crocodiles reared in water that is obtained from a putatively polluted River, expressed CYP isoenzyme activities and proteins with corresponding high liver burden of PAHs in both male and female crocodiles. Inducible CYP1A enzymes are known to exhibit metabolic preference to planar PAHs and in mammals, these isoenzyme are induced by 3-methycholantrene (3-MC) and related compounds [32–34]. Despite the fact that PAH concentrations were similar or even higher in females compared to males, the male hepatic EROD and BROD activities, and CYP1A and CYP3A proteins were higher. These results might be related to differences in individual hydrocarbons (i.e. benzo[a]anthracene was higher in males, SM2), or suggest that expression of CYP isoenzymes in the Nile crocodiles is also modulated by cellular levels of estrogen-like compounds. In accordance with the present study, catalytic activities of inducible and non-inducible CYP enzymes have been shown to decrease with increasing cellular estrogen levels during sexual maturation in several vertebrate species [10,30,35].

Laboratory studies have confirmed that estrogenic compounds, significantly decrease hepatic CYP1A1 transcriptional levels, with a subsequent decrease in functional (EROD activity and protein levels) products in fish *in vivo* and *in vitro* experiments [36,37]. Several hypotheses have been formulated to explain the hormone-mediated down-regulation of CYP1A1. These include the assumption that steroid hormones and/or metabolites can bind the CYP1A1 protein [38] and consequently inhibit the CYP1A1 protein catalytic activity [30]. The inhibitory effect of hormones is partly mediated through the estrogen receptor (ER) in a process where the ER-E2 complex interferes with the CYP1A1 gene directly or through interaction with the aryl hydrocarbon receptor (AhR), that indirectly regulates CYP1A1 gene expression by binding to the xenobiotic response elements (XRE). Furthermore, it has been shown that estrogens and their mimics may control the recruitment of ER and possibly other co-activators, besides activating the detoxification pathway. We also observed that the female crocodiles used in the present study expressed gonadal estrogen receptor (ERα) significantly higher than the males (Arukwe et al. in prep). Thus, the present study confirmed that crocodiles follow this established mode of mechanism by which estrogenic compounds regulate the CYP system.

The CYP3A enzymes play integral roles in steroid metabolism in lower vertebrates [39,40], and the hydroxylation of steroids by CYP3A often relates to gender [39]. While CYP3A proteins are shown to be constitutively expressed in fish [41], regulated during sexual maturation (with males showing higher protein levels than females) and metabolize endogenous substrates such as testosterone and progesterone at the 6β-position [40,42,43], there is increasing scientific evidence suggesting that the pregnane X receptor (PXR), and other nuclear receptors play integral roles in CYP3A induction. This probably occurs in a unique and complex manner that includes induction by structurally diverse compounds with species differences in induction profiles [44–46]. In the present study, the CYP3A showed a higher protein band intensity in male crocodiles, compared to females and this response paralleled the lower E2 level observed in female crocodiles, compared to males. In fish, it was demonstrated that steroid hormone mimics modulate CYP3A-mediated catalytic activities and mRNA levels in a similar manner as natural steroid hormones [47,48].

Overall, the catalytic assays for EROD and BROD (not PROD and MROD) indicated sex-differences between male and female crocodiles and paralleled immunochemically determined CYP1A and CYP3A protein levels, despite the relatively high PAHs levels in both sexes. Interestingly, plasma E2 levels were higher in males (119 ± 71 pg/ml) than females (79 ± 37 pg/ml), suggesting that other physiological or endogenous mechanisms might be playing a role in regulating the sex-related differences observed in these enzyme activities that are observed in this study. In American alligator, the microsomal activity profiles of these same CYP enzymes showed substrate selectivity towards 3-MC- and phenobarbital (PB)-induced P450s [49,50]. We did not observe immunochemically detectable CYP2E1, CYP2B1/2B2, CYP26 proteins using mammalian antisera, probably due the absence of PB-type inducers in the contaminant profiles measured in the specimens. Previously, immunochemical detectable protein bands were observed in PB exposed American alligator using anti-rabbit and anti-scup CYP2B1 and CYP2B2 antibodies that paralleled BROD and PROD enzymatic activities [49]. Furthermore, the presence of CYP enzymes that have epitope homology with CYP1A, CYP2-4 enzymes in the alligator was reported previously [49]. While these immunochemical cross-reactions indicate the presence of these proteins, it remains to be established whether these CYP forms are alligator orthologues of mammalian enzymes [49]. Our findings are consistent with fish, where there is a gap in knowledge and controversy regarding multiple cytochrome P450 enzymes, compared to mammalian species.

For example, in mammals, there is a clear distinction between CYPs with different substrate specificities for EROD, BROD, MROD and PROD. EROD, BROD and PROD activities show strong relations, suggesting a 3-MC inducible CYP1, CYP2 and/or CYP3 isoenzyme [49]. This assumption is supported by the high liver burden of PAHs, which are 3-MC like CYP inducers. The PROD activity has been detected in several freshwater and marine species [51] and the American alligator [49], whose activity has been attributed to barbiturates, non-planar PCBs and DDT inducible CYP2B isoenzyme. In mammals, BROD activity shows broader substrate specificity anda known marker for CYP1A, 2B and 3A—while in alligators, BROD activity appears to be a better indicator of PB-induced CYP2 family isoforms [49]. Generally, several rat CYPs induced BROD activity and that the CYP form(s) primarily responsible for BROD activity in liver microsomes varied according to how the rats were induced [52]. Thus, similar to other species, the detection and measurement of the activity and protein levels of these CYP subfamilies showed a consistent pattern with CYP isoforms and their related sex-differences in crocodiles, as has been reported in other species.

### Oxidative stress responses

Environmental contaminants or stressors induce an imbalance between ROS production and endogenous antioxidants in exposed organisms, eventually leading to alteration of physiological functions of cellular macromolecules including DNA, protein and lipids [53]. Prooxidant chemicals also activate cellular stress-sensitive signaling pathways for adaptive response purposes and the induction of antioxidant system has been proposed to play a significant role in cell protection from oxidative stress [54]. On the other hand, prolonged or acute oxidative challenge can cause the inhibition of the antioxidants efficiency, thus enhancing the intracellular formation of ROS and onset of oxidative damages [14].

There are only a few studies describing selected antioxidant enzymes in crocodilian species [55–56], and a general up-regulation of this system in the caiman *Caiman yacare* was related to variations in intracellular oxygen metabolism during the transition from embryos/hatchlings to juveniles, but not from juveniles to adults [56]. In this study, levels of several antioxidant responses (GST, CAT, GPx, GR, GSH) were integrated with the analyses of both the total oxyradical scavenging capacity (TOSC) toward different oxyradicals and the content of MDA, a typical by-product of lipid peroxidation. Our results indicated an efficient antioxidant system in the Nile crocodile with levels of antioxidant enzymes comparable or even higher than those reported in *C*. *yacare* or in other aquatic vertebrates [56]. In particular, enzymatic activities of catalase, Se-dependent and total glutathione peroxidases suggest an elevated capability to reduce lipid hydroperoxides and hydrogen peroxide, as further corroborated by the high levels of total oxyradical scavenging capacity toward both peroxyl radicals and hydroxyl radicals. The high antioxidant efficiency in the Nile crocodile might be partly associated to the tolerance toward hypoxia and re-oxygenation conditions, which represent a well-known prooxidant challenge in diving animals [56].

However, the samples analyzed in the present study were also characterized by elevated hepatic concentrations of chemical pollutants, notably heavy metals, aliphatic hydrocarbons, benzene and PAHs. In this respect, the biotransformation pathway of cytochrome P450 represents an important source of oxyradical formation, and close relationships have been described between metabolism of organic xenobiotics and the complex antioxidant system network [14]. The non-significant changes in liver burden of heavy metals between male and female crocodiles, also reflected the level of MT in both sexes. The oxidative effects of PAH metabolism in Nile crocodile would be supported by the generally higher levels of antioxidants in males, paralleling the sex-related differences of biotransformation enzymatic activities (EROD, BROD) and proteins content (CYP1A, CYP3A). The greater prooxidant pressure in male crocodiles was also evidenced by the content of MDA, a marker of lipid peroxidation commonly used to reveal oxidative stress responses [57,58]. Moderate differences between male and female crocodiles were observed also for the peroxisomal AOX activity. A wide class of prooxidant chemicals, including PAHs, modulates peroxisomal proliferation, and closely interacts with the CYP450 biotransformation pathway, and further contributes to enhancing intracellular ROS formation [12–15]. Thus, the analyses of liver antioxidant responses, MDA and peroxisomal proliferation in male and female crocodiles provided an integral overview on the extent of oxidative stress that may be related to chemically mediated production of ROS and damage to cellular functions.

In conclusion, the complex interactions between contaminant levels, biotransformation and oxidative network can be modulated at different levels, from gene transcription to cellular effects [59]. While variations in transcript levels may be considered as biomarkers of exposure, those occurring on the enzymatic activities better reflect a functional effect [13]. In this respect, we can suggest that the Nile crocodiles are experiencing oxidative stress from PAH exposure. Oxidative attack to proteins and impairment of their function may generate the need for increased synthesis and energy-demanding processes, thus reducing the available energy for other key physiological processes [60]. While these findings do not prove cause and effect, it is correlational, warranting systematic and differently designed study to discern cause and effects scenarios. However, it should be noted that Le Croc farm is experiencing significant reproduction and general health problems, due to decreased fertility and hatching of crocodiles (Arukwe et al. in prep). Also, the integrity of membranes is extremely important for cellular energy generation, storage and consumption. Mitochondrial ATP-production by ATPase is dependent on the electrochemical gradient of H^+^ across the inner mitochondrial membrane, which might be impaired by contaminant-induced lipid peroxidation and subsequent leakage of H^+^ [61,62]. Preliminary results indicated a direct relationship between chemical burden (PCBs, metals and PAHs) in the different crocodile organs collected in this study, with changes in membrane lipid composition measured in the same organs for both male and female crocodiles (Arukwe et al. in prep).

## Supporting Information

S1 Dataset Complete chemical analysis dataset. (XLSX)

S1 File Extended methods for chemical analyses and biomarker measurements. (DOC)
