# Supplementary material for: Biotransformation and Oxidative Stress Responses in Captive Nile Crocodile (Crocodylus niloticus) Exposed to Organic Contaminants from the Natural Environment in South Africa
Source: PLoS One. 2015 Jun 18;10(6):e0130002. doi: 10.1371/journal.pone.0130002 (PMC4473036; doi:10.1371/journal.pone.0130002)
Supplement: S1 File — (DOC) [file pone.0130002.s002.doc]

**Extended methods for chemical analyses and biomarker measurement**

*Chemical analyses*

Chemical analyses in crocodile tissues have been determined according to previous methods with slight modifications (Bocchetti et al., 2008; Fattorini et al., 2008; Piva et al., 2011; Regoli et al., 2014).

For trace metals, tissues were dried to constant weight at 60°C and digested under pressure with nitric acid and hydrogen peroxide (7:1) with microwave (Fattorini et al., 2008). Arsenic, cadmium, chromium, copper, iron, manganese, nickel, lead, vanadium, zinc were analyzed by atomic absorption spectrophotometry, with flame (Varian, SpectrAA 220FS) and flameless atomization (Varian SpectrAA 240Z); the mercury content was quantified by cold vapor atomic absorption spectrometry (Cetac QuickTrace M-6100 Mercury Analyzer) (Fattorini et al., 2008).

Aliphatic hydrocarbons were extracted treating tissues (about 3 g, wet weight) with hexane:acetone (2:1) in a microwave (110°C for 25 min, 800 Watt) (Mars CEM, CEM Corporation, Matthews NC). After centrifugation at 3.000 × *g* for 10 min, the supernatants were purified with solid-phase extraction (Phenomenex Strata-X, 500 mg × 6 mL plus Phenomenex Strata-FL, 1000 mg × 6 mL) and then concentrated using a SpeedVac (RC1009; Jouan, Nantes, France) to dryness. Samples were finally recovered with 1mL of pure, analytical GC grade *n*-hexane and analyzed with a gas chromatograph (Perkin Elmer) equipped with an Elite-5 capillary column (30 m × 0.32 mm ID × 0.25 μm-df) and a flame ionization detector. For quantitative determination, the system was calibrated with an unsaturated pair *n*-alkane standard mixture according to EN ISO 9377-3 (Fluka 68281).

For analysis of polycyclic aromatic hydrocarbons (PAHs), about 3 g (wet weight) of tissues were extracted in 10 mL 0.5 M potassium hydroxide in methanol with microwave at 55°C for 20 min (800 Watt) (CEM, Mars System). After centrifugation at 3.000 × *g* for 10 min, the methanolic solutions were concentrated using a SpeedVac and purified with solid-phase extraction (Octadecyl C18, 500 mg × 6 mL, Bakerbond). A final volume of 1 mL was recovered with pure, analytical HPLC gradient grade acetonitrile, and HPLC analyses were carried out in a water and acetonitrile gradient by fluorimetric and diode array detection. The PAHs were identified according to the retention times of an appropriate pure standards solution (EPA 610 Polynuclear Aromatic Hydrocarbons Mix), and classified as low molecular weight (LMW: naphthalene, acenaphthylene, 1-methyl naphthalene, 2-methyl naphthalene, acenaphthene, fluorene, phenanthrene, anthracene) or high molecular weight (HMW: fluoranthene, pyrene, benzo(a)antrhacene, chrysene, 7,12-dimethyl benzo(a)anthracene, benzo(b)fluoranthene, benzo(k)fluoranthene, benzo(a)pyrene, dibenzo(a,h)anthracene, benzo(g,h,i)perylene, indeno(1,2,3,c,d)pyrene).

Polychlorinated biphenyls (PCBs), organo-halogenated pesticides (OCPs), chlorophenols, monoaromatic compounds (BTEX: benzene, toluene, ethylbenzene and xylene congeners) and brominated flame retardants (BFRs) have been determined according to EPA methods 1614, 3550B, 3665A, 3630C, 8020, 8081B, 8082A with slight modifications. About 3 g of wet tissues were extracted with hexane:acetone (2:1) solutions in a microwave (110°C for 25 min, 800 Watt) (Mars CEM, CEM Corporation). After centrifugation at 3.000 × g for 10 min, the supernatants were purified with solid-phase extraction (Phenomenex Strata-X, 500mg, 6mL and Phenomenex Strata-FL, 1000mg, 6 mL), using an automated SPE system (Gilson Aspec GX271, Gilson Middleton, WI, USA) and then concentrated using a SpeedVac (RC1009; Jouan, Nantes, France) to dryness. Samples were finally recovered with 1 mL of pure, analytical GC grade n-hexane and analyzed with a GC-MS system (Varian Saturn 2000 ion trap, Agilent Technologies, Santa Clara, CA, USA) using a GC capillary column Zebron (Zebron ZB-5MS, 30m, 0.25mmID, 0.25µ, Phenomenex), applying three different spit-splitless and oven temperature ramp methods for different class of pollutants: 1): BTEX (benzene, toluene, ethylbenzene and xylene congeners); 2): chlorophenols (2,4-dichlorophenol, 2,4,6-trichlorophenol, pentachlorophenol), OCPs (2,4-dichlorophenol, 2,4,6-trichlorophenol, pentachlorophenol, α-lindane, β-lindane, δ-lindane, γ-lindane, α-chlordane, γ-chlordane, 4,4'-DDD, 4,4'-DDE, 4,4'-DDT, aldrin, dichlorobenzidine, dieldrin, endrin, endrin aldehyde, endrin ketone, hexachlorobenzene, methoxychlor, endosulfan I, endosulfan II, endosulfan sulfate, heptachlor, heptachlor epoxide), chlorobiphenyls and PCBs congeners (2-chlorobiphenyl, 3-chlorobiphenyl, 4-chlorobiphenyl, PCB4, PCB8, PCB11, PCB16, PCB17, PCB18, PCB19, PCB28, PCB38, PCB44, PCB46, PCB52, PCB66, PCB77, PCB81, PCB101, PCB105, PCB118, PCB126, PCB128, PCB138, PCB153, PCB156, PCB169, PCB170, PCB172, PCB180, PCB182, PCB187, PCB192, PCB195, PCB203, PCB206 and PCB209); 3): BFRs, including hexabromocyclododecane (HBCD), tetrabromobisphenol A (TBBPA) and polybromodiphenylethers (PBDE28, PBDE47, PBDE100, PBDE99, PBDE154, PBDE153, PBDE183).

The various compounds were determined by the retention time of analytical pure standard solution mix (Supelco 47505-U, BTEX/MTBE; Supelco 43240-U, DM 471 Phenol mix; NIST 1493, PCBs congeners; Supelco 4-8862, Aroclor mix 2; Fluka 36989, PCBs congeners; Supelco 46845-U, Pesticide 8081; Supelco 4-0008, Hexachlorobenzene; Supelco 4-0026, 3,3’-dichlorobenzidine; AccuStandard M-1614-CSM, PBDEs congeners of primary interest; Fluka 11223, TBBPA; Aldrich 144762, HBCD), comparing the mass spectra with those of the pure standard compounds and also verifying these by means of the NIST Database (NIST/EPA/NIH Mass Spectra Library version 2.0, National Institute for Standard and Technologies, NIST, Gaithersburg, MD, USA).

For all of the chemical analyses, quality assurance and quality control were assured and monitored by processing blank and reference standard materials (NIST-2977, National Institute of Standards and Technology, USA); concentrations obtained from these SRM analyses were always within the 95% confidence intervals of the certified values. The water content in tissues was determined for all the samples, and the concentrations were expressed as ng/g dry weight (dw) for PAHs, PCBs, OCPs, BFRs, while as μg/g dry weight (dw) for aliphatic hydrocarbons (C10-C40) and trace metals.

**References (for Chemical methods):**

Bocchetti, R., Fattorini, D., Pisanelli, B., Macchia, S., Oliviero, L., Pilato, F., Pellegrini, D., Regoli, F. 2008. Contaminant accumulation and biomarker responses in caged mussels, Mytilus galloprovincialis, to evaluate bioavailability and toxicological effects of remobilized chemicals during dredging and disposal operations in harbor areas. Aquat. Toxicol. 89: 257–266.

Fattorini, D., Notti, A., Di Mento, R., Cicero, AM., Gabellini, M., Russo, A., Regoli, F. 2008. Seasonal, spatial and inter-annual variations of trace metals in mussels from the Adriatic Sea: a regional gradient for arsenic and implications for monitoring the impact of off-shore activities. Chemosphere. 72: 1524–1533.

Piva, F., Ciaprini, F., Onorati, F., Benedetti, M., Fattorini, D., Ausili, A., Regoli, F. 2011. Assessing sediment hazard through a weight of evidence approach with bioindicator organisms: A practical model to elaborate data from sediment chemistry, bioavailability, biomarkers and ecotoxicological bioassays. Chemosphere 83: 475-485.

Regoli, F., Pellegrini, D., Cicero, AM., Nigro, M., Benedetti, M., Gorbi, S., Fattorini, D., D’Errico, G., Di Carlo, M., Nardi, A., Gaion, A., Scuderi, A., Giuliani, S., Romanelli, G., Berto, D., Trabucco, B., Guidi, P., Bernardeschi, M., Scarcelli, V., Frenzilli, G. 2014. A multidisciplinary weight of evidence approach for environmental risk assessment at the Costa Concordia wreck: Integrative indices from Mussel Watch. Mar. Environ. Res. in-press http://dx.doi.org/10.1016/j.marenvres.2013.09.016

*Biomarkers analyses*

Ethoxyresorufin *O*-deethylase (EROD) was assayed spectrofluorimetrically as described in Regoli et al. (2005). Samples were homogenized (1:5, w/v) in 100 mM K-phosphate buffer pH 7.5, containing 0.15 M KCl and 1 mM ethylendiaminetetraacetic acid (EDTA). After centrifugation at 12,000×g for 15 min, the resulting supernatants (S12) were immediately used for determination of enzyme activities. Incubations were carried out at 25°C in a final volume of 1 ml containing 100 mM K-phosphate buffer pH 7.5, 4 μM 7-ethoxyresorufin and 0.25 mM NADPH for 5 min, before the addition of 2 ml acetone to stop the reaction. Incubation mixtures as described above, but stopped at time zero were used as blank values and subtracted from the sample fluorescence. Fluorimetric analyses (535/585 nm) were quantified by reference to resorufin standards (0.01–1 μM).

Metallothioneins (MTs) were analyzed after acidic ethanol/chloroform fractionation of tissues homogenates, and spectrophotometric quantification using reduced glutathione (GSH) as standard (Viarengo et al., 1997). The activity of peroxisomal Acyl-CoA oxidase (AOX) was measured with a coupled assay following the production of H_2_O_2_ by the oxidation of dichlorofluorescein-diacetate in the presence of an exogenous horseradish peroxidase (Bocchetti et al., 2006). Antioxidants defenses were measured in mussels digestive glands following standardized assay conditions, at a constant temperature of 18 ± 1°C (Bocchetti et al., 2008; Regoli et al., 2005). Catalase was determined by the decrease in absorbance due to H_2_O_2_ consumption; glutathione peroxidases (GPx) activities were assayed in a coupled enzyme system where β-nicotinamide adenine dinucleotide (NADPH) is consumed by glutathione reductase to convert the oxidized glutathione (GSSG) to its reduced form using hydrogen peroxide or cumene hydroperoxide as substrates, respectively, for the selenium-dependent and for the sum of Se-dependent and Se-independent forms; glutathione S-transferases (GST) were determined following the reaction between GSH and 1-chloro-2,4-dinitrobenzene (CDNB) as substrate; glutathione reductase activity was measured by the oxidation of NADPH during the reduction of GSSG; levels of total glutathione were enzymatically assayed after acidic deproteinization with sulphosalicilic acid. The content of malondialdehyde (MDA) was measured after derivatization with 1-metyl-2-phenylindole and spectrophotometrically determined after calibration against a malondialdehyde standard curve (Gorbi et al., 2013).

The Total Oxyradical Scavenging Capacity (TOSC) was measured by the capability of cellular antioxidants to inhibit the oxidation of α-keto-γ-methiolbutyric acid (KMBA) to ethylene gas in the presence of different forms of oxyradicals, like peroxyl radicals (ROO·) and hydroxyl radicals (HO·) which are artificially generated at constant rate (Regoli and Winston, 1998, 1999). Ethylene formation was determined by gas-chromatographic analyses and TOSC values were quantified from the equation: TOSC = 100-(∫SA/ ∫CA x 100), where ∫SA and ∫CA are the integrated areas calculated under the kinetic curve produced during the reaction course for respective sample (SA) and control (CA) reactions. For all the samples, a specific TOSC (normalized to content of protein) was calculated by dividing the experimental TOSC values by the relative protein concentration contained in the assay and determined by the Lowry method with Bovine Serum Albumin (BSA) as standard (Winston et al., 1998).

**References (for biomarkers)**

Bocchetti, R., Regoli, F., 2006. Seasonal variability of oxidative biomarkers, lysosomal parameters, metallothioneins and peroxisomal enzymes in the Mediterranean mussel Mytilus galloprovincialis from Adriatic Sea. Chemosphere 65, 913–921.

Gorbi, S., Avio, G.C., Benedetti, M., Totti, C., Accoroni, S., Pichierri, S., Bacchiocchi, S., Orletti, R., Graziosi, T., Regoli, F., 2013. Effects of harmful dinoflagellate Ostreopsis cf. ovata exposure on immunological, histological and oxidative responses of mussels Mytilus galloprovincialis. Fish Shellfish Immunol . 35, 941-950.

Regoli, F., Nigro, M., Benedetti, M., Gorbi, S., Pretti, C., Gervasi, P.G., Fattorini,D., 2005. Interactions between metabolism of trace metals and xenobiotics agonists of the Ah receptor in the Antarctic fish Trematomus bernacchii: environmental perspectives. Environ. Toxicol. Chem. 24, 1475–1482.

Regoli, F., Winston, G.W., 1998. Application of a new method for measuring the Total Oxyradical Scavenging Capacity in marine invertebrates. Marine Environmental Research 46(1–5), 439–442.

Regoli, F., Winston, G.W., 1999. Quantification of total oxidant scavenging capacity (TOSC) of antioxidants for peroxynitrite, peroxylradicals and hydroxylradicals. Toxicol. Appl. Pharmacol. 156, 96–105.

Viarengo, A., Ponzano, E., Pondero, F., Fabbri, R., 1997. A simple spectrophotometric method for metallothionein evaluation in marine organisms: an application to Mediterranean and Antarctic mollusks. Mar. Environ. Res. 44, 69–84.

Winston, G.W., Regoli, F., Dugas, A.J., Blanchard, K.A., Fong, J.H., 1998. A rapid gas chromatographic assay for determining oxyradical scavenging capacity of antioxidants and biological fluids. Free Rad. Biol. Med. 24, 480–493.
